# Dietary Influences on the Microbiota–Gut–Brain Axis

**DOI:** 10.3390/ijms22073502

**Published:** 2021-03-28

**Authors:** Thomas M. Barber, Georgios Valsamakis, George Mastorakos, Petra Hanson, Ioannis Kyrou, Harpal S. Randeva, Martin O. Weickert

**Affiliations:** 1 Warwickshire Institute for the Study of Diabetes, Endocrinology and Metabolism, University Hospitals Coventry and Warwickshire NHS Trust, Clifford Bridge Road, Coventry CV2 2DX, UK; t.barber@warwick.ac.uk (T.M.B.); gedvalsamakis@yahoo.com (G.V.); drpetrahanson@gmail.com (P.H.); kyrouj@gmail.com (I.K.); harpal.randeva@uhcw.nhs.uk (H.S.R.); 2 Division of Biomedical Sciences, Warwick Medical School, University of Warwick, Coventry CV2 2DX, UK; 3 Endocrine Unit, 2nd Department of Obstetrics and Gynaecology and Pathology Department, Aretaieion University Hospital, Athens Medical School, 11528 Athens, Greece; mastorakg@ath.forthnet.gr; 4 Aston Medical Research Institute, Aston Medical School, College of Health and Life Sciences, Aston University, Birmingham B4 7ET, UK; 5 Centre for Sport, Exercise and Life Sciences, Faculty of Health & Life Sciences, Coventry University, Coventry CV1 5FB, UK

**Keywords:** gut microbiota, brain, diet, appetite, metabolism

## Abstract

Over unimaginable expanses of evolutionary time, our gut microbiota have co-evolved with us, creating a symbiotic relationship in which each is utterly dependent upon the other. Far from confined to the recesses of the alimentary tract, our gut microbiota engage in complex and bi-directional communication with their host, which have far-reaching implications for overall health, wellbeing and normal physiological functioning. Amongst such communication streams, the microbiota–gut–brain axis predominates. Numerous complex mechanisms involve direct effects of the microbiota, or indirect effects through the release and absorption of the metabolic by-products of the gut microbiota. Proposed mechanisms implicate mitochondrial function, the hypothalamus–pituitary–adrenal axis, and autonomic, neuro-humeral, entero-endocrine and immunomodulatory pathways. Furthermore, dietary composition influences the relative abundance of gut microbiota species. Recent human-based data reveal that dietary effects on the gut microbiota can occur rapidly, and that our gut microbiota reflect our diet at any given time, although much inter-individual variation pertains. Although most studies on the effects of dietary macronutrients on the gut microbiota report on associations with relative changes in the abundance of particular species of bacteria, in broad terms, our modern-day animal-based Westernized diets are relatively high in fats and proteins and impoverished in fibres. This creates a perfect storm within the gut in which dysbiosis promotes localized inflammation, enhanced gut wall permeability, increased production of lipopolysaccharides, chronic endotoxemia and a resultant low-grade systemic inflammatory milieu, a harbinger of metabolic dysfunction and many modern-day chronic illnesses. Research should further focus on the colony effects of the gut microbiota on health and wellbeing, and dysbiotic effects on pathogenic pathways. Finally, we should revise our view of the gut microbiota from that of a seething mass of microbes to one of organ-status, on which our health and wellbeing utterly depends. Future guidelines on lifestyle strategies for wellbeing should integrate advice on the optimal establishment and maintenance of a healthy gut microbiota through dietary and other means. Although we are what we eat, perhaps more importantly, we are what our gut microbiota thrive on and they thrive on what we eat.

## 1. Introduction

Hippocrates, the father of modern medicine, said that all disease originates within the gut. It has taken millennia for the prescience of the great man to truly manifest. Over the last two decades, there has been a transformation in our understanding of the role of the gut and its resident microbiota in health and disease. Far from being simply a system whereby digestion and the absorption of nutrients and water occurs, the gastrointestinal system plays an essential role in the maintenance of health and wellbeing, and a central pathogenic role in the origin of much of the 21st century chronic illness burden. These include atopies, food intolerances, auto-immunities, and chronic inflammatory, cardio-metabolic and neuro-psychiatric conditions [1]. The gut microbiota provides a zeitgeist for the host and plays a central role as a determinant of health or disease; as such, it behaves like an organ in its own right, integral to both the gastrointestinal system and normal physiological function.

The term ‘microbiota’ is an umbrella that incorporates the many prokaryotes (bacteria), eukaryotic microorganisms (such as fungi and protozoans), archaea and viruses that associate with the human body, including the skin, genitourinary tract, respiratory epithelia and the gastrointestinal tract. The human microbiota has been estimated to consist of >30 trillion microbes, a similar order of magnitude to the number of our own cells [2]. The majority of the human microbiota reside within the gut, and whilst occupying the entire gastrointestinal tract, the majority (around 70%) of these microbes exist within the colon [3]. Our gut microbiota co-evolved with us over hundreds of millions of years. Over this unimaginable expanse of evolutionary time, our immune systems developed integrally and intricately in a myriad of ways with our gut microbiota. This inter-dependence between our gut microbiota and our immune system forms a key component of our understanding of the importance of the gut microbiota for the maintenance of health and avoidance of disease. Our immune system depends on our gut microbiota for its normal development, whilst our gut microbiota depend on the tolerability of our immune system for its survival: a truly symbiotic relationship. Indeed, arguably the gut microbiota and immune system are not separate entities, but rather a truly interlinked system, with one depending upon the other [4].

Our understanding of the gut microbiota remains very much in its infancy. There are four major microbial phyla that comprise >90% of the bacteria within the gut microbiota: *Bacteroides*, *Firmicutes*, *Actinobacteria* and *Proteobacteria* [5]. In addition to bacteria, our gut microbiota also consists of viruses, fungi, Amoebozoa and archaea [6]. Certain strains of microbes associate with favourable health, such as *Firmicutes* and *Cytophaga, Flavobacterium* and *Bacteroides* [5], and others associate with chronic illness (e.g., obesity and metabolic dysfunction) [7,8,9,10,11,12]. However, these association data on which much of the current literature is based do not provide insight into causation or pathogenesis of disease. Furthermore, there are clear limitations from focusing on one strain of microbe in isolation from the myriad and countless other microbes within its vicinity, all of which interact with each other and with the host. Based on current data, our notion of a healthy gut microbiota (except for the neonatal period) is one that manifests a rich and diverse array of microbes. Such a scenario appears essential for the normal and healthy development of the immune system. Intriguingly, currently only around 1000 species of human microbiota have been identified [3], suggesting that perhaps the vast majority of the gut microbiota remain unknown to us. Furthermore, we have limited or no knowledge or understanding of the impact on human health of many of the known gut microbiota [13].

In this concise review, we focus on the microbiota–gut–brain axis and explore the complex mechanisms that bi-directionally link the gut microbiota with the brain. We also outline current evidence to support the influence of dietary macronutrients on the gut microbiota.

## 2. The Microbiota–Gut–Brain Axis

Traditionally, we considered the gastrointestinal and central nervous systems as separate entities. Accordingly, we also considered gastrointestinal and neuro-psychiatric disorders as separate from one another, with respective etiopathogeneses in silos. Furthermore, until relatively recently, we thought the main role of the colon was for water absorption. Although we had awareness of the gut as a seething mass of microbes, we understood these microbes as such: a seething mass with little known physiological function or importance, other than being a potential source of life-threatening infection following breach of the wall of the gastrointestinal canal.

Our modern understanding of the gut microbiota places it as a central determinant for health and wellbeing, with much of the modern-day 21st century chronic illness burden linked to dysbiosis within the gut microbiota [14]. Indeed, the gut microbiota is implicated in the pathogenesis of many neuro-psychiatric disorders, including Parkinson’s disease [15], autism spectrum disorder [16], chronic pain [17] and disorders of mood and affect [18]. Based on such compelling data, it is timely to modify our traditional view of the gut and the brain as separate entities and replace this with a model in which there is intricate linkage of the gut and its microbiota with the brain through multiple complex and bi-directional pathways: a microbiota–gut–brain axis. From this modern perspective, health and wellbeing depend upon normal functioning of both the gut microbiota and the brain. Furthermore, any pathology that affects either the gut microbiota or the brain does not occur in isolation, but rather impacts on both systems.

Much of our modern insight into the microbiota–gut–brain axis stems from metabolomics data from rodent-based studies [19]. For example, rodent-based models reveal an association between the gut microbiota and levels of important neurotransmitters within the brain. Germ-free (GF) rodents manifest a reduction in the expression of brain-derived neurotrophic factor (BDNF), primarily within the hippocampus [20]. Conversely, rodent models with healthy gut microbiota show increased expression of BDNF within the brain [21]. Furthermore, the gut microbiota also associate with the levels of neuroreceptors within the brain. The gamma-amino butyric acid (GABA) receptor mediates the effects of the eponymous major inhibitory neurotransmitter in the brain and regulates numerous psychological and physiological processes [1,19], including the pathogenesis of anxiety and depression [19]. In one rodent-based study, compared with control-fed mice, chronic ingestion of *Lactobacillus rhamnosus* (JB-1) resulted in regionally dependent changes in GABA receptor expression within the brain, with increases in GABA receptor expression in cortical regions and reductions in the prefrontal cortex and amygdala [19]. These changes in central GABA receptor expression are associated with reduced anxiety- and depression-related behaviour [19]. Other rodent studies showed an association between the gut microbiota and the expression of other neuroreceptors, such as the N-methyl-D-aspartate (NMDA) receptor (which mediates the effects of the excitatory neurotransmitter glutamate) [21], and the central expression of serotonin receptor 1A [19] and tryptophan [22]. Furthermore, gut microbiota-induced changes in the expression of neuroreceptors often associate with altered emotional behaviours [19,21]. As outlined, compelling evidence links the gut microbiota with brain neurotransmission and neuroreceptors, including emotions and behaviour. Thus, it is important to explore the underlying mechanisms and complex pathways that mediate these effects and provide a foundation for the microbiota–gut–brain axis.

## 3. Mechanisms That Link the Gut Microbiota with the Brain

**Figure 1:**
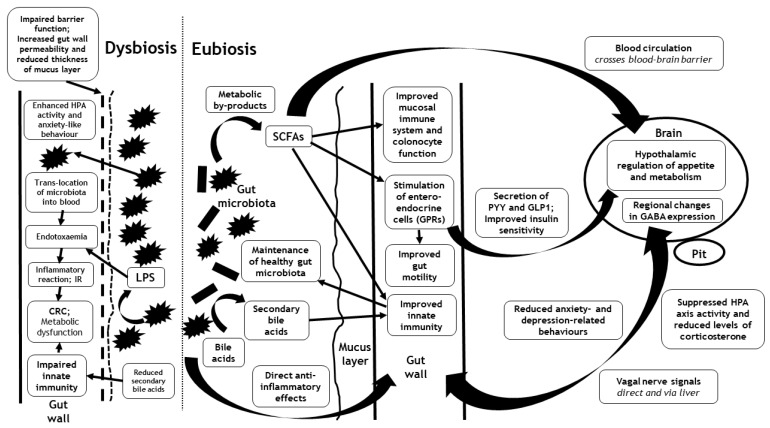
Outline of the major proposed pathways that link the gut microbiota with the brain during eubiosis and dysbiosis, including the hypothalamus–pituitary–adrenal axis (HPA) and autonomic, neuro-humeral, entero-endocrine and immunomodulatory pathways. Interactions may occur directly through translocation of the gut microbiota or their cell walls (endotoxaemia) and/or through the release and absorption of the metabolic by-products of the gut microbiota (such as short chain fatty acids (SCFAs)). Although the interactions between the gut microbiota (and their metabolic by-products) and the brain are mediated via similar pathways in eubiosis and dysbiosis, their effects are opposed. Eubiosis associates with the suppression of the HPA axis and the alleviation of anxiety, whereas dysbiosis associates with enhancement of the HPA axis and anxiety-like behaviour. CRC = colorectal cancer; GABA = gamma-amino butyric acid; GLP1 = glucagon-like peptide-1; GPRs = G protein-coupled receptors; HPA = Hypothalamus-pituitary adrenal; IR = Insulin resistance; LPS = lipopolysaccharide; Pit = pituitary; PYY = peptide YY; SCFA = Short chain fatty acid.

The complex bi-directional pathways that link the gut microbiota with the brain implicate mitochondrial function, the hypothalamus–pituitary–adrenal axis, and autonomic, neuro-humeral, entero-endocrine and immuno-modulatory pathways. Furthermore, direct links between the gut microbiota and the brain may occur through the release and absorption of the metabolic by-products of the microbiota within the gut [23]. In this section, we outline the current evidence to support the major proposed pathways that link the gut microbiota with the brain (summarized in Figure 1).

### 3.1. Metabolic By-Products of the Gut Microbiota and Incretin Hormones

The gut microbiota utilize energy from food products ingested by the host, and in the process release by-products of their metabolism that include short chain fatty acids (SCFAs), secondary bile acids and tryptophan metabolites [19,24]. These metabolic by-products of the gut microbiota likely mediate at least some of the pathways that link the gut microbiota with the brain, either directly or indirectly, through the modulation of signals from enterochromaffin and entero-endocrine cells and the regulation of the mucosal immune system and inflammatory pathways. Interestingly, SCFAs and secondary bile acids also influence host energy production, with implications for athletic performance [25].

Amongst the metabolic by-products of the gut microbiota, there has been much focus on SCFAs, produced by caecal anaerobic microbes, such as *Enterococcus* (a Gram-positive facultative anaerobe of the genus *Lactobacillus* and phylum *Firmicutes*), during the fermentation of dietary fibre (non-digestible carbohydrates). SCFAs (and levels of colonic *Enterococcus*) appear to associate with some beneficial effects to the host, including the inhibition of appetite [26]. Furthermore, SCFAs may cross the intestinal barrier into the systemic circulation, and the blood–brain barrier into the brain parenchyma to exert direct effects on the hypothalamic regulation of metabolism and appetite [27,28]. In addition to direct hypothalamic effects, SCFAs may influence the regulation of metabolism and appetite indirectly through entero-endocrine effects, and glucose, lipid and cholesterol metabolism through effects on G protein-coupled receptors (GPRs) [29]. Expressed within gut adipocytes, immune cells and entero-endocrine cells, there are two SCFA-specific GPRs: GPR41/ free fatty acid receptor 3 (FFAR3) and GPR43/FFAR2 [30]. In a rodent-based study, SCFA stimulation of GPR41 on entero-endocrine cells resulted in enhanced secretion of peptide YY (PYY, a potent appetite-suppressant gut-derived incretin hormone), increased gut motility and reduced harvesting of energy via SCFAs from the diet in wild-type mice vs. GPR41 knockout mice [31]. GPR43 may also mediate SCFA-dependent effects on the optimized release of incretin hormones, such as glucagon-like peptide-1 (GLP-1) and enhanced insulin sensitivity [32]. Finally, SCFAs may provide a useful source of energy for colonocyte function [33].

Some human-based studies also suggest beneficial metabolic effects of SCFAs. One study explored the metabolic effects of the ingestion of a novel inulin-propionate ester (propionate, a common SCFA produced by the human gut microbiota) compared with an inulin control group in overweight adults (n = 60) using a randomized, controlled, crossover design [34]. The ingestion of propionate resulted in an early postprandial release of the incretin hormones, PYY and GLP-1 from human colonic cells, and reduced caloric intake. Furthermore, over 24 weeks of regular propionate ingestion, there was significant weight loss, reduced hepatic lipid content and intra-abdominal adipose tissue volume and preserved insulin sensitivity [34]. These data support an important role for SCFAs in the mediation of the metabolic and appetitive effects of the gut microbiota through entero-endocrine pathways.

Despite some support for the favourable metabolic effects of SCFAs, there remains controversy within the literature regarding the magnitude and nature of such benefits. As metabolic by-products of the gut microbiota, SCFA production depends upon fermentation, a process optimized by the ingestion of the soluble form of dietary fibre. However, our group has demonstrated that the ingestion of insoluble cereal fibres from wheat or oat extracts and whole grain products (demonstrated as non-fermentable *in vivo* and *in vitro* [35]), and not the soluble and highly fermentable types of dietary fibre, associates with improved insulin sensitivity and risk for the development of type 2 diabetes mellitus (T2D) [36]. This is an important observation, which appears to contradict an important role of SCFAs (produced during fermentation) in the mediation of the insulin sensitizing effects of the gut microbiota, as outlined above. A further study from our group compared the metabolic effects of isoenergetic high-protein versus high-cereal fibre diets, randomly assigned to overweight adults with features of metabolic syndrome [37]. After six weeks of each diet, insulin sensitivity significantly improved in those participants assigned to the high-cereal fibre diet compared with those assigned to the high-protein diet [37]. Cereal fibre is insoluble and has a limited capacity for fermentation by the gut microbiota, and therefore limits any attendant production of metabolic by-products, such as SCFAs, from the gut microbiota. Therefore, these data corroborate the view that the improved insulin sensitivity resulting from the ingestion of insoluble cereal fibre, cannot be explained solely through fermentation by the gut microbiota and resultant SCFA production, and alternate explanations are required [37]. Furthermore, although the intake of dietary fibre can influence serum levels of PYY [38], this does not necessarily translate into improved satiety or metabolic benefits [39]. Despite these caveats and controversies, SCFAs probably contribute up to 10% of the energy we extract from our food [40,41,42,43]. Future studies should explore the effects of SCFAs on other incretin hormones known to affect appetite regulation, such as glucose-dependent insulinotropic polypeptide (GIP) [44,45,46].

The gut microbiota also bio-convert bile acids (synthesized from cholesterol within the liver) into secondary bile acids. This bioconversion is important, as secondary bile acids contribute towards innate immunity, insulin sensitivity and optimal regulation of host metabolic pathways (including carbohydrate and lipid metabolism) through the modulation of signalling pathways via the G protein-coupled membrane receptor 5 (TGR5) and the nuclear farnesoid X receptor (FXR) [47]. Furthermore, secondary bile acids, through modulation of the intestinal innate immune response, also ensure the maintenance of a healthy gut microbiota [47,48,49]. Dysbiosis within the gut microbiota associates with the aberrant bioconversion of bile acids into secondary bile acids, which in turn results in both metabolic dysfunction and gastrointestinal carcinogenesis (including colorectal cancer and hepatocellular carcinoma) [48].

Succinate is another metabolic by-product of the gut microbiota and is produced by colonic bacterial species such as *Prevotellaceae* and *Veillonellaceae* [14]. Similar to SCFAs, succinate also crosses the intestinal wall and appears within the circulation. However, succinate associates unfavourably with health. Indeed, serum succinate levels associate with obesity and metabolic syndrome, and conversely weight loss associates with a reduction in serum levels of succinate and an increase in the proportion of succinate-consuming colonic bacteria, including *Odoribacteraceae* and *Clostridiaceae* [50]. Unlike SCFAs however, any potential role for succinate in mediating links between the gut microbiota and the brain remains unidentified. Succinate may simply represent an inert serum biomarker of succinate-producing colonic microbiota, without any causative role in the pathogenesis of disease. Future research should explore a possible role for succinate in the mediation of the microbiota–gut–brain axis.

### 3.2. Mitochondrial Function

There are complex bi-directional interlinks between the gut microbiota and host mitochondrial function [14] which are influenced by genetic variants within the mitochondrial genome [25]. The gut microbiota regulate key enzymes, transcription factors and transcriptional co-activators involved in mitochondrial biogenesis in the host. Mitochondrial function (including the mitochondrial production of reactive oxygen species) influences the host response to the gut microbiota (including mucosal immune responses and intestinal barrier function), and in turn helps to regulate the gut microbiota [14]. The development of autism spectrum disorder possibly implicates mediating effects of mitochondrial function induced by colonic butyrate [14]. Neuronal mitochondrial dysfunction (affecting neuronal function and neuron cell numbers) may mediate the effects of gut dysbiosis on depression [51]. Possible pathways include SCFAs, brain inflammatory processes following gut permeability and increased blood lipopolysaccharide (LPS) levels [51].

### 3.3. Hypothalamus–Pituitary–Adrenal (HPA) Axis

Rodent-based studies suggest a link between the gut microbiota and activity within the HPA axis. Germ-free (GF) mice manifest enhanced basal or stimulated HPA axis activity [19], whereas in normal mice, probiotics induced suppression of HPA axis activity (reflected by reduced levels of corticosterone) [19]. It has been hypothesized that increased HPA axis activity in GF mice may reflect a loss of microbiota-related energy sources [1]. Interestingly, although some controversy exists regarding the concordance of anxiety-like behaviours and the activity of the HPA axis, in one study, GF mice did manifest both increased HPA axis responsiveness and anxiety-like behaviour [52]. The gut microbiota influence the activity of the HPA axis through several mediators that cross the blood–brain barrier. These include cytokines, prostaglandins and microbial antigens [53]. Furthermore, activation of the HPA axis may influence the gut microbiota and intestinal permeability [53]. Severe mental disorders such as bipolar disorder, schizophrenia and depression associate with dysbiosis, intestinal permeability and dysregulation of the HPA axis [53]. Although complex bi-directional pathways exist, dysbiosis may predispose individuals to mental disorders through multiple mechanisms that include HPA axis dysregulation [53].

### 3.4. Autonomic Signals

Compelling rodent-based data promote an important role for the autonomic nervous system in the mediation of signalling between the gut microbiota and the brain. In one study by Bravo and colleagues, chronic ingestion of a *Lactobacillus* strain associated with regional changes in GABA expression within the brain, reduced stress-induced HPA axis activity and anxiety- and depression-related behaviours. However, these effects did not occur in vagotomised mice [19]. Therefore, the vagus nerve appears to play a central role in mediating bi-directional signals between the gut microbiota and the brain. Some of the autonomic signalling between the gut microbiota and the brain likely implicate the liver, with bi-directional signalling between the gut microbiota and the liver having an impact on feeding behaviour and metabolic control [54].

### 3.5. Immuno-Inflammatory Pathways

Given the intricate links between the gut microbiota and the host immune system, it is perhaps not surprising that the immuno-inflammatory pathway/system represents a highly plausible means by which communications manifest between the gut microbiota and the host. Indeed, certain species of gut microbiota associate with changes in the inflammatory milieu of the host. One such example is the Gram-positive anaerobe *Faecalibacterium prausnitzii*, which associates with anti-inflammatory effects within the host. Possible mechanisms include the blockade of nuclear factor-κB activation with subsequent inhibited secretion of pro-inflammatory mediators [55,56,57]. The mechanisms by which Gram-positive anaerobes, such as *Faecalibacterium prausnitzii,* confer anti-inflammatory and metabolic benefits for the host may occur indirectly, through for example the production of metabolic by-products such as butyrate (a key SCFA). Indeed, there is evidence to support an association between gut microbiota-derived butyrate and improved energy metabolism in rodents [58]. Neuro-inflammation stimulated by translocated bacteria (in the context of dysbiosis and intestinal permeability), including activation of the innate immune system, may represent an important contributor to the pathogenesis of certain neurological and psychiatric conditions such as schizophrenia and other psychotic disorders [59].

### 3.6. Gut Wall Integrity

The gut wall forms the boundary between the gut microbiota and the host. As such, complex bi-directional communication between the gut microbiota and the host brain must either cross (or at least influence in some way) the gut wall. Such communications may occur either directly through the translocation of the microbiota (or some microbiota component) and/or their metabolic by-products, or indirectly through effects via the host immuno-inflammatory, autonomic or endocrine systems. Fascinatingly, the integrity of the gut wall may influence the inflammatory processes within the brain and neuropathology following traumatic brain injury (TBI) [60]. Bi-directional pathways exist in which structural and functional damage to the GI tract can occur following a head injury, which in turn influence the progression of neuropathology and neurodegeneration, including the development of post-TBI chronic traumatic encephalopathy [60]. The integrity of the gut wall may therefore represent a therapeutic target to mitigate the adverse neuropathological outcomes following TBI, to reduce the risk of development of psychiatric disease and psychosis and to promote healthy mental and emotional functioning.

Notably, there is protection of the gut wall itself by a layer of mucus (mucin) that acts as a first line of defence. Chassaing and colleagues explored the thickness of the colonic wall mucus layer in a human-based study by measuring the distance between the gut microbiota and the gut epithelial lining on colonic biopsies [61]. Interestingly, there was an inverse correlation between colonic mucus thickness and the metabolic measures of body mass index (BMI), HbA1C and fasting glucose levels [61]. Although causality cannot be proven, these data are consistent with the notion that mucus-mediated gut wall permeability influences overall metabolic health. Furthermore, gut microbiota play an important role in influencing the colonic wall mucus layer. For example, *Akkermansia muciniphila* is a mucin-degrading bacterium that may influence the colonic wall mucus layer [62]. Clarke and colleagues demonstrated a higher proportion of *Akkermansia* species within the gut microbiota of professional rugby players and controls who were also athletes and had a low BMI, compared with the gut microbiota from high BMI controls [63]. In a different study, levels of colonic *Akkermansia muciniphila* were reduced in adults with obesity and T2D [64]. The example of *Akkermansia muciniphila* is useful to illustrate the perils of considering one species of gut microbiota in isolation from the myriad of others. *Akkermansia muciniphila* degrades mucin, and therefore acting in isolation, it would likely impair the essential mucin colonic epithelial protective barrier [65,66,67]. However, *Akkermansia muciniphila* also converts intestinal mucin to propionic and acetic acid [68], and thereby engages in a symbiotic relationship with the host to provide essential nutrients that are accessible to other resident gut microbiota within the vicinity [64,69]. Many of these other microbiota may in turn have protective effects on the colonic mucin layer and impact on other microbiota and the host through a myriad of mechanisms [70]. Through such insights, future models of the gut microbiota and its interactions with the host should incorporate a more holistic perspective, with the promotion of interactions within the gut microbiota and between the gut microbiota and the host, rather than too much focus on the effects of individual species of microbiota on the host considered in isolation. It is only through the development of such complex models that we will truly understand the intricacies of how the gut microbiota interacts with itself and with its host.

To summarize this section, the microbiota–gut–brain axis forms an essential component for metabolic and overall health and wellbeing. The mechanisms implicated are complex and bi-directional and include effects of metabolic by-products of the gut microbiota, the incretin system, mitochondrial function, the HPA axis, autonomic signals, immuno-inflammatory pathways, liver signalling and gut wall integrity. Much of the current literature relies on association-based studies, rodent models and study of the effects of individual species of gut microbiota on the host in isolation. As such, the field of gut microbiota and its host interactions is in its infancy. We need to move beyond simply labelling species within the gut microbiota as ‘good’ or ‘bad’. Rather, we need to develop a more sophisticated model whereby there is deserved consideration of the gut microbiota as a colony that interacts en masse with its host. Such a model would act as an expedient to the development of effective lifestyle measures to optimize the establishment and maintenance of a healthy gut microbiota, with health-promoting effects. Amongst such lifestyle measures, our diet plays an essential role, and so it is important to consider dietary effects on the gut microbiota.

## 4. Dietary Influences on the Gut Microbiota

Although multiple lifestyle factors contribute towards the establishment and maintenance of the gut microbiota, diet plays a major role. To corroborate this view, a fascinating archaeological study on teeth revealed significant changes in the human gut microbiota during periods of rapid dietary change amongst our human ancestors [71]. This included the transition from the hunter-gatherer Palaeolithic to the farming Neolithic eras around 10,000 years ago (with the adoption of a high-carbohydrate diet), and the beginning of the industrialized era around 200 years ago (with the adoption of a diet rich in processed flour and carbohydrates) [71]. Since the dawn of the industrialized era, Western diets have further changed, with a substantial reduction in the ingestion of dietary fibre derived primarily from unprocessed plant-based foods, mirrored by a reciprocal increase in the ingestion of ultra-highly processed foods that are often sterile, heavily laden with fats and carbohydrates and impoverished of dietary fibre [72]. Rodent-based studies reveal that changes in dietary macronutrient intake can consistently alter the gut microbiota within a single day [73]. Although many previously reported dietary studies in human cohorts had timeframes of weeks or months [74], with changes in a limited number of species within the gut microbiota [75,76] or failure to demonstrate any significant diet-induced changes in the gut microbiota [77], more recent compelling data reveal changes in the gut microbiota composition resulting from short-term dietary changes [78]. In one of the most deeply phenotyped studies reported to date using metagenomic sequencing, there were significant associations between gut microbes and specific nutrients and food groups, driven particularly by healthy and diverse plant-based foods [79]. Furthermore, overall microbiome composition was predictive for multiple cardio-metabolic blood markers, suggesting the potential for future stratification of the gut microbiota as a predictor of future health and illness prior to the development of clinically manifesting disease [79].

Dietary studies in human participants are inherently difficult to conduct for a variety of reasons that include limitations in the accurate recording of dietary intake and the study of macronutrient changes in isolation from the rest of the diet and other lifestyle factors. Furthermore, diet-induced changes in the gut microbiota do not necessarily translate into changes in brain functioning or other host-related effects. Despite these caveats, it is important to consider the evidence for how individual macronutrients influence the human gut microbiota, including dietary fibres (plant-based diets) and dietary fats (animal-based diets), summarized in Table 1.

### 4.1. Dietary Fibres

Dietary fibres comprise two main groups: (i) complex carbohydrates (including digestible and non-digestible forms), and (ii) oligosaccharides [5]. Dietary fibres have an important influence on the composition of the gut microbiota and its fermentative metabolism [5]. Perhaps more than any other macronutrient, dietary fibres play an important role in the establishment and nurture of a healthy gut microbiota and the promotion of health and wellbeing [72].

### 4.2. Complex Carbohydrates

The ingestion of complex carbohydrates promotes bacterial growth favourable for health within the gut microbiota, including *Bifidobacteria* species (*Bifidobacterium breve*, *Bifidobacterium longum* and *Bacteroides thetaiotaomicron*) [80]. Whilst the ingestion of dietary fibre as complex carbohydrates normally includes a combination of both digestible (soluble) and non-digestible (insoluble) types, each has specific effects on the gut microbiota. The ingestion of digestible fibres associates with an increase in the proportion of *Bacteroides* species and butyrate-producing bacteria, such as *Eubacterium rectale* and *Clostridium leptum* [81,82]. Only a fraction of the diet remains undigested as complex carbohydrates when it reaches the colon, including plant cell wall polysaccharides, cellulose and resistant starches [83]. Using an anaerobic *in vitro* continuous flow system and faecal samples from human participants, Leitch and colleagues showed that the ingestion of non-digestible resistant starches was associated with an increased abundance of *Ruminococcus* species, *Bifidobacterium adolescentis*, *Eubacterium rectale* and *Roseburia* species [84]. An important insight from this study is that specific subsets of bacteria are likely the primary colonisers of particular insoluble substrates within the colon, but that the primary colonising species for each substrate may differ between each individual host [84]. Furthermore, data from a human-based study showed that resistant starches with a chemical cross-linking configuration influenced phylum-level changes within the gut microbiota, including an increase in the proportion of *Bacteroides* and *Actinobacteria*, and a reduction in the proportion of *Firmicutes* [85]

The ingestion of dietary fibre associates with health and wellbeing. The role of the gut microbiota in the mediation of the numerous health benefits of dietary fibre remains incompletely understood [72]. However, clear evidence implicates an important role for ingested non-digestible fibre in the derivation of certain nutrients and energy harvesting mediated by the gut microbiota in concert with intestinal digestive enzymes. These nutrients and released energy are then utilised by the host and other resident gut microbiota. Some examples of such gut microbiota-derived nutrients include vitamins like folic acid, biotin and pantothenate that are synthesized by *Bacteroides*, *Eubacterium*, *Fusobacterium* and *Propionibacterium* [86]. Of note, some species of gut microbiota compete with their host for certain nutrients. One example of such competition is *Bacteroides thetaiotaomicron*, which utilises vitamin B12 for its own needs. In one human-based study, a surface-exposed lipoprotein (BtuG) in *Bacteroides thetaiotaomicron* bound with great affinity to B12, with sequestration of B12 from intrinsic factor, thereby reducing the availability of vitamin B12 for absorption and utilisation by the host [87]. However, as outlined earlier, it is important to consider the impact of the gut microbiota on nutrient availability and energy harvesting from a colony derived perspective rather than the effects of individual species of microbiota in isolation. What is incontrovertible is that despite some competition with the host, the gut microbiota provides us with essential nutrients, without which our health would suffer. Furthermore, the ingestion of dietary fibre, particularly in its non-digestible form, provides an essential ingredient for this process to occur naturally.

### 4.3. Oligosaccharides

In addition to complex carbohydrates, the ingestion of oligosaccharides also influences the gut microbiota. In one microarray analysis, the ingestion of fructan was associated with a reduction in both *Clostridium* and *Bacteroides* species [88]. In another human-based study, fructan ingestion promoted the growth of butyrate-producing bacteria, such as *Faecalibacterium prausnitzii* [89]. Conversely, the ingestion of inulin and fructo-oligosaccharides appears to promote the growth of *Lactobacillus* and *Bifidobacterium* species [88]. Finally, human-based data reveal that the ingestion of galacto-oligosaccharides can stimulate the growth of *Faecalibacterium prausnitzii* and species of *Bifidobacteria* [90].

### 4.4. Dietary Fats

When considering the effects of dietary fat on the gut microbiota, it is important to clarify the type of model used (human vs. rodent) and the type of fatty acid assessed: (i) saturated fatty acid (SFA); (ii) monounsaturated fatty acid (MUFA); or (iii) polyunsaturated fatty acid (PUFA), of which there are two types (ω-6 and ω-3) [5,91]. It is much easier to regulate the dietary constituents of SFA, MUFA and PUFA in rodent models than in humans. Accordingly, most of the reported studies on the effects of these specific types of fatty acids on the gut microbiota stem from murine models. These include murine-based diets rich in SFAs that associate with the growth of delta-*Proteobacteria*, including *Bilophila wadsworthia* [92], and those high in ω-6 PUFAs that associate with a reduction in the populations of *Bacteroides* whilst enriching populations of *Firmicutes*, *Proteobacteria* and *Actinobacteria* [93]. Indeed, murine-based studies also reveal that a high-fat diet results in dysbiosis, with a significant reduction in the numbers of *Roseburia* species [94].

Regarding human-based studies, Wu and colleagues demonstrated that a high-fat diet over a longer term was positively associated with an abundance of *Bacteroides* and *Actinobacteria*, but negatively associated with *Firmicutes* and *Proteobacteria* [77]. Interestingly, a high-fibre diet manifested opposite effects on the gut microbiota to those shown with a high-fat diet [77]. In a further human-based study, a high intake of MUFA associated with higher populations of *Bacteroides* and lower levels of *Bifidobacteria* species, whilst high dietary intake of ω-6 PUFA also associated with a reduction in the population of *Bifidobacteria* [82].

Human-based high-fat diets tend to derive from animal-based diets (including meats, eggs and cheeses). One study reported on the rapid effects of predominantly plant-based and animal-based diets (with an abundance of fibre and fats, respectively) on the gut microbiota. Human participants (n = 10) consumed each diet *ad libitum* for a period of five days [78]. For the high-fat, animal-based diet, data revealed a rapid change in the β-diversity of the gut microbiota (a measure of the difference between baseline and diet-associated gut microbiota) a single day after the diet reached the distal gut microbiota. This included significant changes in the abundance of 22 clusters of gut microbiota species [78]. Fascinatingly, the most abundant taxon amongst these clusters (including *Bacteroides*, *Alistipes* and *Bilophila*) exhibited bile-resistance, consistent with the association of high fat intake with the enhanced secretion of bile acids [78,95]. Furthermore, in addition to changes in β-diversity, there were also alterations in microbial metabolic activity for each diet, with the animal-based diet associated with a significant reduction in products of carbohydrate fermentation and an increase in the products of amino acid fermentation [78]. Following the end of the animal-based diet, the gut microbiota reverted to its original structure within two days [78].

To summarize this sub-section, data from both rodent- and human-based studies reveal a myriad of influences of our diet on our gut microbiota. Although there are some clear health benefits from the ingestion of certain macronutrients (such as non-digestible fibre) and the derivation of essential nutrients, much of the available data simply report on associations of certain macronutrients with changes in the relative growth and abundance of particular bacterial species within the gut microbiota. However, some evidence extends beyond mere association between diet and gut bacterial species. An important example stems from a mouse model that reveals an association between the consumption of a high-fat diet with colonic inflammation. The underlying pathogenic pathway possibly involves the secretion of bile acids, which in turn promotes the growth of sulphite-reducing bacteria such as *Bilophila wadsworth*; the release of hydrogen sulphide then causes inflammation of the intestinal mucosa [92]. Interestingly, human-based studies also reveal an association between a high-fat diet and low-grade inflammation within the gut, including the promotion of the growth of *Bilophila wadsworth* [78].

In addition to localized inflammation, it is also important to consider the effects of dietary macronutrients on gut permeability. In this regard, *Bifidobacteria* species appear to protect the permeability of the gut wall through improved barrier function and reduced production of intestinal LPS [96]. Conversely, the promotion of certain Gram-negative bacteria, such as *Enterobacteriaceae*, through high-fat diets can increase intestinal levels of LPS [97]. LPS can activate Toll-like receptor 4 (TLR-4) signalling, which is implicated in the pathogenesis of glioblastoma multiforme [98]. Furthermore, the ingestion of high-protein diets can promote a localized inflammatory response within the gut, resulting from the production of toxic metabolites stimulated by certain bacterial enzymes [99]. Therefore, from a holistic perspective, typical Western diets (which are high in both protein and fats and impoverished of fibre) promote intestinal dysbiosis with consequently impaired protection of gut wall permeability and a localized inflammatory response [100]. The combination of increased gut wall permeability, colonic inflammation and enhanced colonic production of LPS results in chronic endotoxaemia with excessive bacterial wall LPS present within the circulation. This, in turn, provokes systemic low-grade inflammation and ultimately metabolic dysfunction that underlies much of the modern-day chronic illness burden [101].

## 5. Conclusions and Future Directions

We can perhaps think of the gut microbiota as the last ‘organ’ discovered in the human body. There are, of course, many reasons why the gut microbiota would not fit our conventional view of an organ. Firstly, the gut microbiota technically reside outside of the body, and therefore from a purist perspective, we cannot consider this as part of the body as such. Furthermore, the gut microbiota are composed of prokaryotic cells that are alien, albeit very distantly related, to the host. Perhaps our main objection though is that organs have a supremely refined, highly ordered and organized structure that enables optimal performance of some vital function. The gut microbiota, in contrast, are perhaps the antithesis to what one would define as ‘highly ordered and organized’. However, if we re-define an organ as a collection of cells that fulfils some vital role for the body and which communicates with other organs through physiological pathways, the gut microbiota has as much right to be on that list as any other conventional organ. The gut microbiota indeed fulfils functions that are vital for health and wellbeing, and dysbiosis underlies many chronic illnesses through important and complex pathogenic pathways [102]. As such, the gut microbiota are central to normal physiological function [103] and should, in our view, be considered as an organ in its own right, albeit an externalised organ and one formed of foreign cells. As with any organ, functionality only becomes apparent when considered as a whole. Just as focused attention on a single cardiomyocyte provides little insight into the function of the heart, so too does attention on a single species from the gut microbiota limit our understanding of the functioning of the gut microbiota as a whole colony. Similarly, just as the cells within an organ function together as a group to benefit the entire organism, so too do individual microbiota within the gut interact as a colony in complex ways, both with each other and with the host. Therefore, future studies should focus more on the colony derived effects of the gut microbiota both within itself and with the host.

Our co-evolution with our microbial and viral environments has, in some cases, transitioned beyond mere symbiosis to unity. Examples include the evolution of the eukaryotic cell with the origin of mitochondria, and the striking observation that between 5–8% of the human genome derives from viral sequences similar to infectious retroviruses [104]. However, our relationship with our gut microbiota remains symbiotic, with a key role for the brain. From an evolutionary perspective, we can understand the relevance of the mechanisms that interlink the brain and the gut microbiota [105]. Given the central control of appetite, key metabolic processes and eating behaviours [106], it is no surprise that elements of the microbiota–gut–brain axis feature prominently amongst the identified gut microbiota–host interactions. In short, our gut microbiota have co-evolved with us to manipulate our brains to their own advantage, and vice versa. Elucidation of the actual mechanisms implicated, and the influence of both microbiota- and host-related factors, remains an important challenge for the future.

Although numerous lifestyle factors, including sleep, physical activity and stress, may influence the gut microbiota in important ways [107,108], it is beyond the scope of this concise review to provide such details. Instead, we focus on the effects of dietary macronutrients on the gut microbiota. Diet is inherently difficult to study in humans for a variety of reasons, not least due to the difficulty of studying the effects of a single macronutrient in isolation, and problems with the accuracy of self-recall regarding dietary intake [72]. Furthermore, evidence to support clear effects of a particular macronutrient on the gut microbiota signature does not prove any potential downstream effects on the host. Future studies should explore the effects of dietary and other lifestyle factors on not just individual microbes within the microbiota, but the microbiota colony as a whole, in addition to the interactions of the gut microbiota with the host. Such studies will provide clear insights into how best to optimise our own diets and lifestyles to establish, maintain and nurture a healthy gut microbiota. These insights will provide a basis for future guidelines on healthy living and healthy ageing.

Our modern-day diets are vastly different from what our hominid ancestors would have eaten [109]. For one, ultra-highly processed foods have only been available to us very recently [110], and is something that our gut microbiota have never experienced previously. Furthermore, our modern-day world, including our food, is highly sterilized compared to the evolutionary norm. Whilst such sterilization has helped to address infections and infestations, our gut microbiota have also needed to adapt rapidly. It is possible that a reduction in the replenishing effects of ingested microorganisms (present within natural food sources), may be harmful to us. Indeed, in the study outlined by David and colleagues, bacteria in common fermented foods from both plant- and animal-based diets (such as lactic acid bacteria within cheese and cured meats), reached the gut following ingestion, and were detectable on sequencing analysis [78]. Therefore, the introduction of foreign microorganisms within our food may represent an important means by which our gut microbiota addresses its constant need for replenishment.

Finally, in our quest to explore the gut microbiota and its effects on us, it is important to consider its modification through means alternate to dietary change, such as faecal transplantation. Currently, faecal transplantation has only one indication within the National Health Service (NHS) in the UK: in the management of patients with intractable colonic colonisation with *Clostridium difficile* [111]. Evidence from murine models reveals that faecal transplantation can result in changes in both body weight and metabolic status [112]. Human studies on faecal transplantation show improvements in glucose tolerance but have not yet demonstrated effective weight loss in recipients with obesity [112,113]. The inherent complexity of the gut microbiota, its interaction with the host and its uniqueness to each individual pose significant challenges for its investigation. Rodent-based studies provide compelling data and proof-of-concept that modification of the faecal microbiota translates into metabolic transformation within the host. Whilst caution is required in the translation of rodent to human-based data, such evidence should enthuse and inspire us to explore the therapeutic potential of the manipulation of the gut microbiota in humans to optimize health and wellbeing through both dietary and faecal modifications.

## Figures and Tables

**Table 1:** Influence of dietary macronutrients (including plant-based dietary fibre and animal-based dietary fat) on the gut microbiota, with changes in bacterial groups and their biological significance. LPS = Lipopolysaccharide; SCFA = Short Chain Fatty Acid

Dietary Macronutrient	Nutrient Subtype	Diet Type	Bacterial Changes	Biological Significance
Dietary fibre: complex carbohydrate	Digestible (soluble) fibre	Plant-based	Increased: *Bacteroides* species, *Eubacterium rectale* and *Clostridium leptum*	Promotion of insulin sensitivity
Dietary fibre: complex carbohydrate	Non-digestible resistant starch	Plant-based	Increased: *Ruminococcus* species, *Bifidobacterium* *adolescentis*, *Eubacterium rectale* and *Roseburia* species	Energy harvesting and derivation of essential nutrients such as folic acid, biotin and pantothenate
Dietary fibre: complex carbohydrate	Non-digestible resistant starch with a chemical cross-linking configuration	Plant-based	Increased: *Bacteroides* and *Actinobacteria* species Reduced: *Firmicutes*	
Dietary fibre: oligosaccharides	Fructan	Plant-based	Increased: *Faecalibacterium prausnitzii* Reduced: *Clostridium* and *Bacteroides* species	Increased levels of SCFAs including butyrate; protection of the permeability of the gut wall; reduced production of intestinal LPS; anti-inflammatory effects
Dietary fibre: oligosaccharides	Inulin and fructo-oligosaccharides	Plant-based	Increased: *Lactobacillus* and *Bifidobacterium* species	
Dietary fibre: oligosaccharides	Galacto-oligosaccharides	Plant-based	Increased: *Faecalibacterium prausnitzii* and *Bifidobacterium* species	
Dietary fat	Saturated fatty acids	Animal-based	Increased: *Bilophila wadsworthia*	Inflammation of intestinal mucosa from the release of hydrogen sulphide and the secretion of bile acids
Dietary fat	ω-6 Polyunsaturated fatty acids	Animal-based	Increased: *Firmicutes*, *Proteobacteria* and *Actinobacteria* species Reduced: *Bacteroides* and *Bifidobacterium* species	Impaired barrier function of the gut wall with increased permeability; increased intestinal production of LPS and endotoxaemia
Dietary fat	Monounsaturated fatty acids	Animal-based	Increased: *Bacteroides* species Reduced: *Bifidobacteria* species	
